# Spatially-informed insights into early marriage and school dropout: An advanced bivariate binary multilevel model for understanding Ethiopia's context

**DOI:** 10.1016/j.heliyon.2024.e32005

**Published:** 2024-05-28

**Authors:** Kassie Wubet Ebabu, Demeke Lakew Workie, Ashenafi Abate Woya, Teshager Assefa Sisha

**Affiliations:** a Department of Statistics, Assossa University, Ethiopia; b Department of Statistics, Bahir Dar University, Ethiopia; c Department of Agricultural Economics, University of Kentucky, USA

**Keywords:** Early marriage, School dropout, Bivariate binary multilevel, Ethiopia

## Abstract

The phenomenon of school dropout, which entails the failure to meet the minimum educational requirements, and early marriage, which involves the marital union of girls prior to attaining 18 years of age, constitute crucial issues in Ethiopia. This research endeavor sought to identify the determinants of these two outcomes. A weighted sample of 3091 girls who had experienced early marriage and school dropout was drawn from the 2016 Ethiopian Demographic and Health Survey (EDHS) dataset and analyzed utilizing bivariate binary multilevel models featuring spatial effects. The prevalence rates of early marriage and school dropout were 62.9 % and 75.4 %, respectively. We observed non-uniform spatial distributions of early marriage and school dropout across Ethiopia. The odds ratio of the association between early marriage and school dropout was 1.39, indicating a significant interdependence of these two outcomes. The probability of early marriage and school dropout was estimated to be 1.63 and 1.18 times higher, respectively, for girls hailing from rural areas and 1.70 and 1.23 times higher, respectively, for those classified in the poorest wealth index, as compared to their counterparts. Therefore, stakeholders and policymakers must prioritize hotspots, socio-economic, and demographic factors to achieve a meaningful reduction in the incidence of early marriage and school dropout.

## 1 Introduction

The phenomenon of school dropout indicates a failure to meet basic educational requirements, whereas early marriage involves girls getting married before they turn 18 years of age. Once married, young girls face significant challenges in pursuing both formal and informal education due to household duties, childbirth, and the belief in some societies that marriage and schooling cannot go hand in hand [1]. Millions of girls in Ethiopia have been forced into early marriages in recent times, severely limiting their fundamental rights to life, freedom, education, and other essential needs [2,3].

In addition to causing school dropout, early marriage also has negative implications for women's health. According to a World Health Organization (WHO) report, globally, thousands of women die due to early pregnancy and childbirth, and around 70,000 early-married women aged between 15 and 18 die each year due to pregnancy and childbirth complications [4]. To attain eight out of the seventeen sustainable development goals pertaining to maternal health and well-being by 2030, it is imperative to eradicate early marriage, notwithstanding its widespread occurrence. While Ethiopia has made significant progress in terms of school enrollment and attainment (e.g., primary school enrollment rate increased from 88 % in 2009/10 to 104 % in 2019/20) [5], girls in rural areas are still dropping out of school at the age of 15 years [3].

Early marriage has been found to have an inverse relationship with education, maternal health, poverty reduction, and other pro-women (employment, participation in leadership and politics etc.) concerns [6]. Such gender inequality leads to a significant impact on girls' education. One in four girls globally misses out on secondary school. Since 2007, sub-Saharan Africa has seen a rise of 7 million out-of-school girls due to population growth [7]. Worldwide, over 57 million primary-aged children are out of school in 2017, with over half of them in sub-Saharan Africa [7].

Early school dropout is often cited as one of the most harmful effects of early marriage and motherhood for females in developing nations. Once a girl is married, she is likely to be excluded from school [8,9]. Dropout rates are estimated to be higher in South and West Asia (43 %) and sub-Saharan Africa (36 %), including Ethiopia [10].

The prevalence of early marriage and school dropout varies significantly in Ethiopia, from region to region and from urban to rural areas. Focusing on changes in the prevalence of child marriage in Ethiopia between 2005 and 2016, Erulkar [11] reported a decline in the percentage of young women married before age 18 from 49 % to 40 %. Despite this reduction, the figures still highlight a significant prevalence of child marriage in the country. Primary school dropout rate increased from 12.4 % in 2006/07 to 14.6 % in 2007/09. The primary completion rate during this period stagnated around 44 %, indicating persistent challenges in the Ethiopian education system regarding dropout and completion rates [12]. As of the 2019/20 academic year, the dropout rate for grades 1–8 stood at 14 %, with the highest incidence of dropout recorded in grade 1 at 22 % [13]. As a result, Ethiopia remains one of the countries with the highest rates of early marriage and school dropout [3,14].

Cultural and societal nuances contributing to the prevalence of early marriage include traditional beliefs [15], economic factors [16], gender inequality [17], and lack of education [1]. Traditional beliefs often prioritize early marriage to uphold family honor or adhere to religious customs. Economic pressures may drive families to marry off daughters early as a means of alleviating financial strain or securing economic stability. Gender norms and power dynamics limit girls' autonomy and decision-making power, reinforcing the practice of early marriage [18]. Additionally, communities with limited access to education may view marriage as a primary role for women, relegating educational pursuits to a secondary priority. These factors intertwine to perpetuate early marriage practices in various cultural contexts.

Studying the phenomenon of early marriage and school dropout among girls yields significant policy implications that can inform targeted interventions and policy strategies. Addressing these challenges is crucial for promoting gender equality, advancing educational opportunities, and fostering socio-economic development. By understanding the factors contributing to early marriage and school dropout, policymakers can implement initiatives aimed at preventing these outcomes and supporting the education and empowerment of girls. Policies focusing on improving access to education, raising awareness about the harmful effects of early marriage, and providing support services for at-risk girls can help mitigate the prevalence of these practices. Additionally, investments in community-based programs, parental education, and economic empowerment initiatives can create an enabling environment that promotes girls' rights and opportunities for a brighter future [19].

Although several studies have been conducted in Ethiopia to identify the determinants of early marriage and school dropout among women separately [20,21], these studies did not consider the spatial effects, which limits the provision of a consistent estimate and valid statistical inference [22]. Given the high rates of early marriage and school dropout worldwide, it is essential to study their causes and consequences closely.

Therefore, we aim to identify factors that influence early marriage and school dropout among girls in Ethiopia, using bivariate binary multilevel models with spatial effects from the Ethiopian Demographic and Health Survey (EDHS) 2016 data, considering Ethiopia's administrative zones as spatial variation. Thus, the primary strength of the model used in this study was its ability to account for the dependency of outcomes, weighted sampling, and spatial effects to obtain consistent estimates.

To the best of our knowledge, this paper represents the first comprehensive analysis of the effects of various covariates on early marriage and school dropout using a bivariate binary multilevel model and a large dataset. Identifying specific predictors associated with early marriage and school dropout is crucial for prioritizing interventions aimed at achieving a substantial reduction in these rates.

## 2 Data and methods

**Fig. 1:**
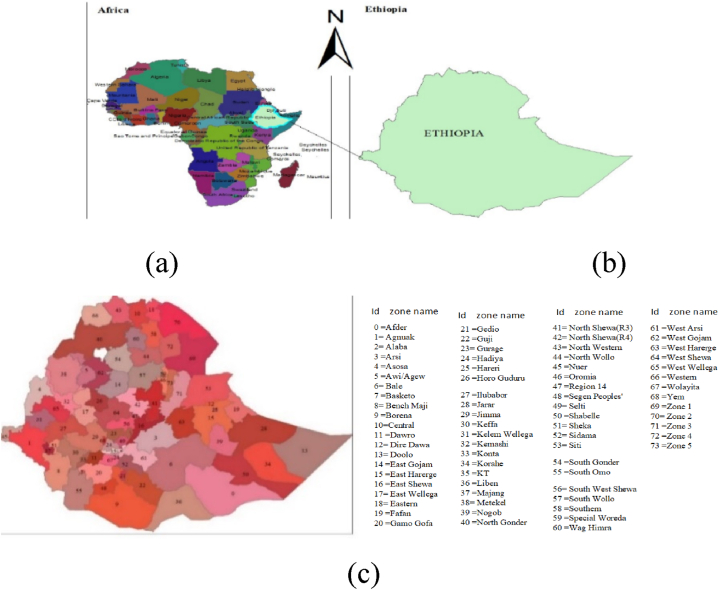
Map of Africa (a), Ethiopia with regions (b) & administrative zones (c) Source: Ethiopian central statistical authority (CSA).

The study was conducted in Ethiopia, which is situated in the Horn of Africa (Fig. 1a). Ethiopia is divided into nine regions and two city administrations (Fig. 1b), which are further subdivided into 74 zones (Fig. 1c).

We utilized data from the 2016 EDHS, which is publicly available at https://www.dhsprogram.com. The surveyed sample was meticulously crafted to ensure representation from both urban and rural areas, reflecting the diversity of settings in the country. The first stage involved the selection of a total of 645 clusters, of which 202 were in urban areas and 443 were in rural areas. Cluster selection was carried out using probability proportional to cluster size. In the second stage, a systematic sampling approach was employed to select 28 households per enumeration area (EA), where an EA is defined as a geographic area demarcated for the purposes of the census. The selection of households was based on an equal probability systematic selection approach, from a household list newly created for this purpose. By adopting this meticulous two-stage approach, the survey aimed to capture the nuances and variations inherent in both urban and rural environments, thereby enhancing the comprehensiveness and validity of the findings [23].

For this study, all eligible women aged 15–49 years who were early married and had dropped out of school within five years prior to the survey were included. In Ethiopia, the concept of ‘school age’ for enrollment does not hold as rigidly as in some other places [24]. It is common to encounter students who are older than the typical ‘school age’ yet are still actively pursuing education. Thus, there is not always a direct alignment between the traditional notion of school age and school attendance in Ethiopia. Therefore, we permit our sample to include respondents who are significantly above the typical ‘school age’. The final sample size consisted of 3091 such women, weighted to account for the survey design. The study aimed to include all married women aged 15 to 49 who resided in the enumeration area during the 5 years preceding the survey. However, to ensure data quality and accuracy, certain exclusions were applied. Specifically, women who had missing values in their response variables, as well as those whose age was below 15 or above 49, or who were not married, were excluded from the analysis. Moreover, to control for spatial effects, zones with longitude and latitude values of zero degrees were excluded from the study. These measures were taken to enhance the internal validity of the study and ensure that the results obtained are robust and reliable.

A bivariate binary multilevel model was used to analyze both early marriage and school dropout following previous studies by Ref. [25]. Bivariate regression models are used to analyze the relationship between two dependent variables that are correlated, considering other covariates as influencing factors in the model. This approach is preferred over separate models as it produces more consistent estimates and less standard error, resulting in more reliable inferential statistics [26]. More specifically, bivariate binary logistic regression analysis was employed to model the two binary dependent variables jointly as a function of covariates [27,28]. This is because the outcomes are often correlated, and the odds ratio is a natural measure for the association between two binary variables [28].

Early marriage is any marriage entered into before one reaches the legal age of 18 [29] whereas school dropout has been defined as leaving education without obtaining a minimal credential, most often a higher secondary education diploma [9]. Therefore, this study has two binary outcomes. The response variable for the *i*^*t*ℎ^ married women in *j*^*t*ℎ^ cluster is represented by a random variable *Y*_*ij*_ with two possible values coded as 1 and 0. That is the first response variable was early marriage and coded as:

$$Y_{\text{ij}}=\left\{ \begin{array}{c} 1,\text{if}\;\text{age}\;\text{at}\;\text{first}\;\text{marriage}\;\text{is}\;\text{below}\;18\;\text{years}=\text{Yes}\;\\ 0,\text{if}\;\text{age}\;\text{at}\;\text{first}\;\text{marriage}\;\text{is}\;18\;\text{and}\;\text{above}\;\text{years}=\text{No} \end{array}\right.$$

and the second response variable of this study was school dropout and coded as:

$$Y_{\text{ij}}=\left\{ \begin{array}{c} 1,\text{if}\;\text{woman}\;\text{dropout}\;\text{from}\;\text{school}=\text{Yes}\;\\ 0,\text{if}\;\text{woman}\;\text{did}\;\text{not}\;\text{dropout}\;\text{from}\;\text{school}=\text{No}\; \end{array}\right.$$

Therefore, bivariate binary logistic regression analysis has two binary outcomes (Y_1_, Y_2_) and four possible joint probabilities as presented in equation (1).

**Table:** 

		**School dropout (**$Y_{2}$**)**		Total
		0	1	
**Early marriage (**$Y_{1}$**)**	0	$\pi_{00}=p\left(Y_{1}=0,Y_{2}=0\right)$	$\pi_{01}=p\left(Y_{1}=0,Y_{2}=1\right)$	${1-\pi }_{1}$
	1	$\pi_{10}=p\left(Y_{1}=1,Y_{2}=0\right)$	$\pi_{11}=p\left(Y_{1}=1,Y_{2}=1\right)$	$\pi_{1}$
	Total	$1-\pi_{2}$	$\pi_{2}$	1

Thus, the bivariate binary logistic model with a function of predictors is expressed as:

(1) $$\begin{array}{rr} p_{1}\left(x\right) & =\text{logit}\;\left[\pi_{1}\left(x\right)\right]=\log\;\left[\frac{\pi_{1}\left(x\right)}{1-\pi_{1}\left(x\right)}\right]=x^{T}\beta_{1}\\ p_{2}\left(x\right) & =\text{logit}\;\left[\pi_{2}\left(x\right)\right]=\log\;\left[\frac{\pi_{2}\left(x\right)}{1-\pi_{2}\left(x\right)}\right]=x^{T}\beta_{2}\\ p_{3}\left(x\right) & =\log\;\left[\psi \left(x\right)\right]=\log\;\left[\frac{\pi_{11}\left(x\right)\pi_{00}\left(x\right)}{\pi_{10}\left(x\right)\pi_{01}\left(x\right)}\right]=x^{T}\beta_{3} \end{array}$$

where, β_1_ and β_2_ refers to the parameters of the conditional logit model for Y_1_ = 1 and Y_1_ = 0, respectively in a function of predictors, X; β_3_ = β_1_ - β_2_; π1(*x*) and π_2_(*x*) are marginal probabilities of outcomes, and ***ψ***(*x*) is the odds ratio of outcomes depending on covariates, which shows the dependency between the outcomes [28,30]. Finally, the bivariate binary multilevel logistic model with auto-covariate (a covariate that captures the dependence between observations within the same cluster or group) variable is expressed in equation (2):

(2) $$Y_{ijk}=\sum_{p=1}^{2}d_{pijk}\left[\Upsilon_{op}+\sum_{s=1}^{S}\Upsilon_{sp}x_{sjk}+pS_{i}+\sum_{s=1}^{S}u_{spjk}x_{sjk}+u_{pjk}+v_{pk}\right]$$

where d_pijk_ is denote a dummy variable for the two responses assuming the value 1 when p = i and 0 otherwise; $S_{i}$ is the auto-covariate variable and $u_{pjk}$ and $v_{pk}$ are intercept and slope variations, respectively. All the logistic models of the parameters are estimated by the maximum likelihood techniques and individual parameter estimates are tested using the Wald statistic [31]. The goodness-of-fit test of the model was checked.

The selection of specific predictors is guided by theoretical principles, drawing upon prior research that identifies factors influencing the early marriage and school dropout rates among girls (Table 1). In addition, place of residence, region, and spatial auto-covariate variable ($S_{i}$) are considered as community level predictors.

**Table 1:** The association of predictors with school dropout conditioned on early marriage.

Predictors	Non-dropped & Non-married n (%)	Non-dropped & Married n (%)	Dropped & Non-married n (%)	Dropped & Married n (%)	P-value
Age in year					0.866
<20	10(3.0)	31(3.4)	85(21.9)	218(16.7)	
20-34	264(78.6)	701(77.3)	203(52.3)	776(59.3)	
35-49	62(18.5)	175(19.3)	100(25.8)	315(24.1)	
Religion					0.000
Orthodox	208(61.9)	358(39.5)	228(58.8)	476(36.4)	
Catholic	0(0.0)	11(1.2)	3(0.8)	9(0.7)	
Protestant	80(23.8)	282(31.1)	82(21.1)	350(26.8)	
Muslim	45(13.4)	252(27.8)	74(19.1)	458(35.0)	
Others	3(0.9)	4(0.4)	1(0.3)	14(1.1)	
Women education					0.000
No education	3(0.9)	20(2.2)	10(2.6)	39(3.0)	
Primary	71(21.1)	626(69.0)	185(47.7)	1138(87.0)	
Secondary	104(31.0)	200(22.1)	128(33.0)	103(7.9)	
Higher	158(47.0)	61(6.7)	65(16.8)	28(2.1)	
Husband education					0.000
No education	13(4.3)	138(17.1)	31(10.1)	266(23.7)	
Primary	54(18.0)	376(46.7)	85(27.8)	617(55.0)	
Secondary	68(22.7)	198(24.6)	96(31.4)	166(14.8)	
Higher	165(55.0)	93(11.6)	94(30.7)	72(6.4)	
Husband occupation					0.040
Not working	19(6.3)	49(6.1)	24(7.8)	104(9.3)	
Working	282(93.7)	756(93.9)	282(92.2)	1016(90.7)	
Media exposure					0.000
No	33(9.8)	389(42.9)	101(26.0)	604(46.2)	
Yes	304(90.2)	518(57.1)	287(74.0)	704(53.8)	
Wealth index					
Poorest	7(2.1)	67(7.4)	25(6.4)	137(10.5)	0.000
Poorer	3(0.9)	129(14.2)	24(6.2)	237(18.1)	
Middle	22(6.5)	182(20.1)	41(10.6)	256(19.6)	
Richer	50(14.9)	212(23.4)	65(16.8)	300(22.9)	
Richest	254(75.6)	317(35.0)	233(60.1)	379(29.0)	
Decision to marry					0.000
Myself	245(72.9)	502(55.4)	201(51.8)	510(39.0)	
Parents	80(23.8)	354(39.1)	182(46.9)	698(53.4)	
Others	11(3.3)	50(5.5)	5(1.3)	100(7.6)	
Distance to health Facility					0.000
No problem	291(86.6)	246(27.1)	55(14.2)	402(30.7)	
Big problem	45(13.4)	661(72.9)	333(85.8)	906(69.3)	
Ethnicity					0.000
Amhara	130(38.7)	210(23.2)	162(41.6)	352(26.9)	
Oromo	86(25.6)	331(36.5)	80(20.6)	464(35.4)	
Tigrie	41(12.2)	79(8.7)	13(13.6)	85(6.5)	
Affar	1(0.1)	1(0.1)	3(0.8)	6(0.5)	
Somalia	6(1.8)	7(0.8)	11(2.8)	13(1.0)	
Others	72(21.4)	279(30.8)	80(20.6)	389(29.7)	
Place of residence					
Urban	244 (72.9	254 (28.0)	210 (54.1)	280 (21.4)	0.000
Rural	92 (27.4)	653 (72.0)	178 (45.9)	1028 (78.6)	
Region					
Tigry	31 (9.2)	74 (8.2)	48 (12.4)	79 (6.0)	0.000
Afar	2 (0.6)	2 (0.2)	6 (1.6)	6 (0.5)	
**Amhara**	**74 (22.0)**	**98 (10.8)**	**122 (31.5)**	**245 (18.7)**	

$S_{i}$ is a weighted average of the geographic units among a set of neighbors of the geographic unit i and may be calculated as $S_{i}=\frac{\sum_{j=1}^{k_{i}}w_{ij}y_{j}}{\sum_{j=1}^{k_{i}}w_{ij}}$ where y_j_ is the proportion of the outcomes of interest at site j and w_ij_ is kxk weighted matrix that measure the intensity of the relationship among pairs of spatial units denoted as 1 if i and j are neighbors and 0 otherwise [32,33]. While, adding a weight matrix function of neighboring proportion values to the model as explanatory variable result in model improvements by increased predictive accuracy and model versatility.

**Fig. 2:**
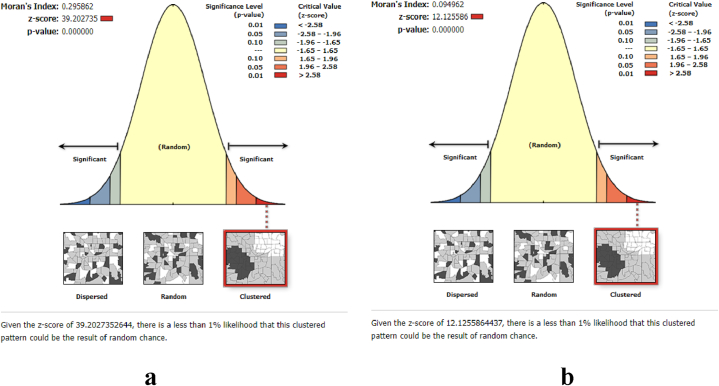
Spatial autocorrelation analysis for early marriage (**a**) and school dropout (**b**).

Finally, we utilized *Moran's I* index to detect spatial clustering and recognized the importance of incorporating spatial elements in our analysis for precise results (Fig. 2). To analyze spatially varying data accurately and meaningfully, statistical methods need to account for the spatial arrangement and correlations among observations [34,35]. Our study analyzes spatial autocorrelation [34,36], hot spot and cluster analysis [37], spatial interpolation [38] and spatial effect analysis [33,39].

with administrative zones of women in Ethiopia from EDHS-2016.

## 3 Results

**Fig. 3:**
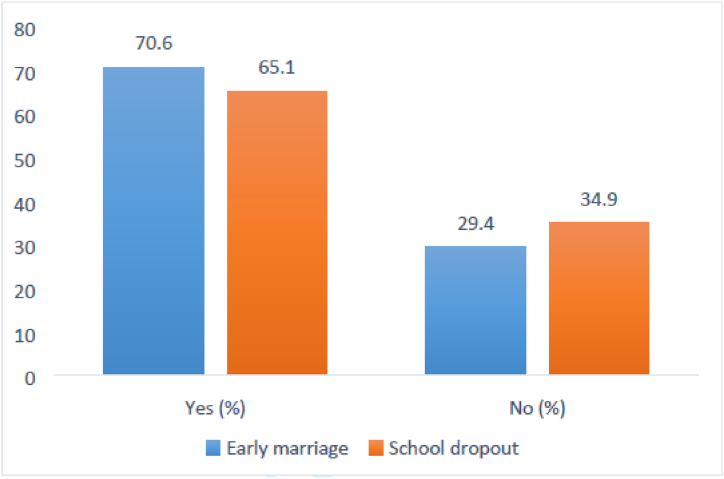
The percentages of early marriage (blue), and school dropout (orange) of women in Ethiopia from EDHS-2016.

This study analyzed 3091 weighted samples of early marriage and school dropout among girls. Exploratory analysis was presented in various forms, including Fig. 3 and Table 1. Fig. 3 displays the description and frequency of outcomes, revealing that 2182 (70.6 %) girls were married early (before the age of 18) and 2011 (65.1 %) dropped out of school after getting married. Table 1 provides a summary of the predictors used in the analysis.

Table 1 presents the results of the chi-square (χ2) test analyzing the association between covariates and early marriage and school dropout. The table shows that several individual predictors were statistically associated with the outcomes, including religion, educational level of girls, husband educational level, husband occupation, media exposure of girls, wealth index, decision to marry, distance to health facility, and ethnicity.

Table 2 presents the joint and marginal probabilities of early marriage and school dropout, along with their odds ratio. The odds ratio is a commonly used measure of the association between two binary responses, with a value of unity indicating statistical independence. However, when the odds ratio (1.649) deviates from unity, as in this case, accounting for the possible dependency between the two outcomes using a bivariate binary logistic regression model is a natural choice.

**Table 2:** Joint and marginal probability of early married and school dropout.

		School Dropout		Margin of early married	Odds Ratio
		Yes	No		
Early Married	Yes	1308(0.445)	388(0.132)	1696(0.577)	1.249
	No	907(0.309)	336(0.114)	1243(0.423)	
Margin of school dropout		2215(75.40)	724(24.60)	2939(1.00)	

Furthermore, the random effect variances of early marriage (0.393; p-value = 0.001), school dropout (0.518; p-value = 0.001), and their covariance (0.071; p-value = 0.001) were found to be statistically significant. This suggests that a bivariate binary multilevel logistic regression model is appropriate.

**Fig. 4:**
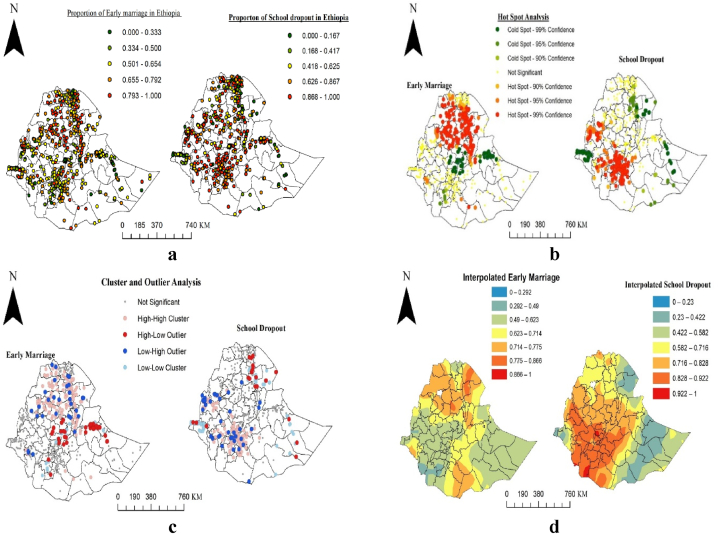
(a) Spatial distribution (b) hot and cold spot analysis (c) cluster and outlier analysis (d) kriging interpolation of early marriage and school dropout of girls in Ethiopian administrative zones from EDHS-2016 dataset.

Fig. 4a displays the spatial distribution of early marriage and school dropout among girls in the administrative zones of Ethiopia. Each point on the map represents the proportion of girls who were married early or dropped out of school, with red indicating the highest proportion and green indicating the lowest. Fig. 4b shows the Local Getis-Ord Gi* statistics, revealing significant hotspot (where the rates of early marriage and school dropout is significantly higher than what would be expected by chance) and coldspot (an area where the observed outcomes are significantly lower than expected by chance) areas for early marriage and school dropout in the administrative zones of Ethiopia. Red indicates areas with a high risk of early marriage and school dropout, while green indicates low risk areas. As such, the northern part of Ethiopia was identified as a high-risk area for early marriage, while the southern and western parts were identified as high-risk areas for school dropout after marriage. In Fig. 4c, red indicates a high rate of early marriage and school dropout surrounded by low rates, while blue shows a low rate of early marriage and school dropout surrounded by high rates. Fig. 4d illustrates the predicted high-risk areas of early marriage and school dropout in red and low-risk areas in blue. The estimated Global Moran's I for early marriage (0.296; p-value = 0.0001) and school dropout (0.095; p-value = 0.0001) were statistically significant. Therefore, a spatial auto-covariate variable was included in the bivariate binary logistic regression model.

**Fig. 5:**
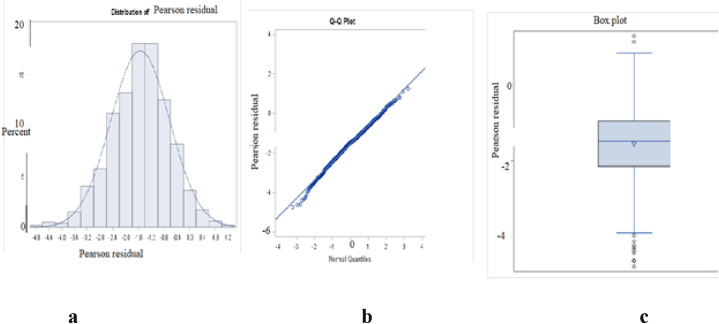
Pearson residuals histogram (**a**), Q-Q plots (**b**) and box plot (**c**) for bivariate binary multilevel with spatial effect logistic regression model.

Several models were fitted to the data, including a random intercept model with level-1 predictors (AIC = 6228.3), a random intercept and random slope model with level-1 predictors (lack of convergence), a random intercept model with level-1 and level-2 predictors (AIC = 6208.3), and a random intercept model with cross-level interaction (AIC = 6301.3). Based on the smallest AIC value, the random intercept model with level-1 and level-2 predictors was found to be the best fit. Additionally, the assumptions of linearity and normality of residuals for the selected model were approximately satisfied, as shown in Fig. 5.

The results of the bivariate binary multilevel analysis presented in Table 3 demonstrate the effects of socio-demographic characteristics on the likelihood of early marriage and school dropout. The analysis indicates that several variables were statistically significant predictors for both early marriage and school dropout simultaneously. Specifically, wealth index, decision to marry, auto-covariate variable, and place of residence were found to be significant predictors for both outcomes. In contrast, husband occupation, girls’ education level, and ethnic group of the respondent were significant predictors for early marriage, while husband education, age of respondent, and media exposure were significant predictors for school dropout.

**Table 3:** Results of bivariate binary multilevel logistic regression model.

Intercept	Early marriage			School dropout		
	Estimate	Std. error	P-value	Estimate	Std. error	P-value
	−1.125	0.644	0.082	−0.928	0.634	0.145
Age						
<20	−0.292	0.175	0.090	−0.546**	0.165	0.001
20-34	0.216	0.117	0.060	−0.246*	0.110	0.026
Religion						
Catholic	0.303	1.005	0.762	−1.195	0.890	0.183
Protestant	−0.284	0.214	0.188	0.047	0.230	0.838
Muslim	0.387	0.974	0.692	1.113	1.215	0.362
Girls Education						
No education	1.753**	0.352	0.000	0.202	0.345	0.558
Primary	1.369**	0.342	0.000	0.198	0.336	0.555
Secondary	0.165	0.347	0.634	0.284	0.339	0.403
Husband education						
No education	0.061	0.278	0.828	0.049*	0.280	0.027
Primary	0.002	0.272	0.993	−0.142	0.276	0.607
Secondary	0.118	0.280	0.675	−0.308	0.282	0.861
Husband occupation						
Not working	−0.408*	0.176	0.022	0.171	0.179	0.342
Media Exposure						
No	0.104*	0.117	0.037	0.030*	0.114	0.027
Wealth index						
Poorest	0.530*	0.252	0.036	0.204*	0.246	0.040
Poorer	0.607*	0.255	0.018	0.067	0.245	0.786
Middle	0.448	0.256	0.080	0.133	0.243	0.583
Richer	0.532*	0.251	0.035	0.038*	0.239	0.038
Decision to marry						
Myself	−0.281	0.423	0.516	1.271**	0.408	0.002
Parents	0.473*	0.419	0.026	1.053**	0.389	0.007
Place of residence						
Rural	0.489*	0.251	0.006	0.162*	0.276	0.043
Ethnic group						
Afar	0.369	0.318	0.248	−0.127	0.332	0.702
Oromo	1.062*	0.454	0.021	0.445	0.434	0.307
Others	0.092	0.560	0.869	0.177	0.548	0.747
Tigrie	−0.072	0.407	0.859	0.324	0.408	0.428
Auto covariate		0.211*	0.736	0.043	−2.314**	0.886
Measure of dependence (OR) = 1.393						
Intercept variance for early marriage	0.213		0.006	0.532		0.011

Key: ** (significant at p-value ≤0.001); * (significant at p-value <0.05).

Furthermore, the estimated odds ratios reveal that early marriage was more prevalent among girls who lived in rural areas and identified as Oromo ethnicity, as the odds of early marriage for these girls were 1.63 $\left(e^{0.489}\right)$ and 2.90 $\left(e^{1.062}\right)$ times higher than those for girls living in urban areas and identifying as Amhara, respectively. This indicates that the estimated odds of early marriage were 63.1 % and 89.2 % higher for girls in rural areas and of the Oromo ethnic group, respectively, than for girls in urban areas and of the Amhara ethnic group in the same clusters. Moreover, the analysis shows that girls from poorer households were more likely to marry early than those from wealthier households, as the estimated odds of early marriage for girls in the poorest, poorer, and richer wealth index families were 1.70 $\left(e^{0.530}\right)$, 1.80 $\left(e^{0.607}\right)$, and 1.70 $\left(e^{0.532}\right)$ times higher than those for girls in the richest wealth index, respectively.

The education level of girls and their husbands also played a significant role in early marriage. The estimated odds of early marriage were 5.78 ($e^{1.753}$) and 3.93 ($e^{1.369}$) times higher for girls who did not attend education and attended primary education, respectively, than for girls who attended higher education in the same clusters. Girls who had uneducated husbands were less likely to marry early than those with educated husbands, as their estimated odds ratio was 0.66 $\left(e^{-0.408}\right)$. Conversely, girls whose marriage decision was carried by their parents were more likely to marry early, as their estimated odds ratio was 1.61 $\left(e^{0.473}\right)$. Finally, the auto-covariate variable was found to be positive (0.21) and significant (p−value=0.043), indicating that zones with a high proportion of early marriage were typically surrounded by zones with a high proportion of early marriage.

The results of the bivariate binary logistic regression analysis showed that the estimated odds of girls living in rural areas and having no media exposure having dropped out from school after getting married were 1.18 $\left(e^{0.162}\right)$ and 1.03 $\left(e^{0.030}\right)$ times more likely than the estimated odds of girls living in urban areas and having exposure to mass media, respectively. These results indicate that the estimated odds of girls living in rural areas and having no exposure to mass media dropping out of school were higher by 17.6 % and 3.0 % than the estimated odds of girls living in urban areas and having exposure to mass media in the same clusters, respectively. This suggests that girls living in urban areas and having exposure to mass media in the same clusters were less likely to drop out of school than girls living in rural areas and having exposure to mass media.

Additionally, the estimated odds of school dropout of girls in the poorest and richer wealth index after getting married of the same clusters were 1.23 $\left(e^{0.204}\right)$, and 1.04 $\left(e^{0.038}\right)$ times higher than the estimated odds of girls in the richest wealth index, respectively. These findings indicate that the estimated odds of girls in the poorest and richer wealth index being dropouts from school after early marriage were 22.6 % and 3.9 % higher than the estimated odds of girls in the richest wealth index of the same clusters.

The estimated odds of school dropout of girls whose marriage decision made by themselves and by their parents were 3.56 $\left(e^{1.271}\right)$ and 2.87 $\left(e^{1.053}\right)$ times higher than the estimated odds of girls whose marriage decision made by others in the same clusters, respectively. Similarly, the estimated odds of school dropout of girls whose age was less than 20 and 20–34 were 0.79 and 0.58 times lower than the estimated odds of girls whose age was 35–49, respectively. The estimated odds of school dropout after marriage of girls having an uneducated husband were 1.05 $\left(e^{0.049}\right)$ times more likely than the estimated odds of girls compared with the reference group. These results suggest that girls who made their own marriage decision or had younger ages were less likely to drop out of school, while girls whose marriage decisions were made by others or had older ages were more likely to drop out of school after marriage. Furthermore, girls who had uneducated husbands were more likely to drop out of school after marriage compared to the reference group. The spatial autocorrelation variable (auto covariate) was negative (−2.314) and significant (p value = 0.009), indicating that zones with a low proportion of school dropouts were usually surrounded by zones with a high proportion of school dropouts. Finally, the significant random components of the model showed that both early marriage and school dropout had a significant variance at the cluster level.

## 4 Discussions

The findings of this study were consistent with previous research conducted in Ethiopia, which also utilized bivariate binary models to analyze related issues such as child composite index anthropometric failure and household wealth index [28], as well as modern contraceptive use and knowledge of HIV/AIDS prevention [31]. Based on data from the EDHS in 2016, it was verified that the prevalence of early marriage among girls aged 15–49 in Ethiopia was 62.9 %, showing a slight decrease from 64.2 % reported in the EDHS 2011 [20]. A comparison with other countries revealed that the prevalence of early marriage in Ethiopia was higher than in Nepal (52 %), India (41 %), and Pakistan (37 %) [40]. This difference can be attributed to various factors such as economic disparities, cultural variations, and differences in educational opportunities between Ethiopia and these countries [1,15,16,21].

Spatial analysis identified several high-risk areas for early marriage, including North and South Wollo, Zones one, two, and four of Afar, North and South Gondar, West and East Gojam, Awi/Agaw, Wag Himra, Western, Southern, Eastern, and Northwestern Tigray, Guji, and Shaka. These findings were consistent with a study conducted by Agerenehu et al. [41], which also identified these regions as hotspots for early marriage [41]. Additionally, the study found that the prevalence of school dropout after marriage in Ethiopia was 75.4 % among girls aged 15–49, based on EDHS 2016 data. High-risk areas for school dropout included Dawro, West Arsi, Gamo Giffa, Gurage, Assosa, West Wellega, Wolayita, Alaba, Hadiya, KT, Sidama, Metekel, West Shewa, Kemashi, and Arsi.

The multilevel logistic regression model revealed several statistically significant predictors for both early marriage and school dropout, including the decision to marry, place of residence, autocovariate, and wealth index. Media exposure, respondent age, and husband's educational level were found to be significant predictors for school dropout, while girl's educational level, husband's occupation, and ethnic group were significant predictors for early marriage. The study confirmed that girls living in rural areas were more likely to experience early marriage and school dropout compared to girls in urban areas. These findings were consistent with previous research conducted in Ethiopia [20,21,42,43], and Turkey [44] on the determinants of early marriage and school dropout.

The study also highlighted the influence of parental decision-making on early marriage and school dropout. Girls whose parents made the decision to marry them were more likely to experience early marriage and dropout from school compared to girls who had a say in their own marriage. This can be attributed to parents not considering the age and educational aspirations of their daughters when making marriage decisions [45].

Furthermore, the study found that wealth index played a significant role in early marriage and school dropout. Girls from the poorest wealth index category were more likely to experience early marriage and school dropout compared to girls from the richest wealth index category within the same clusters. These findings were consistent with previous studies conducted in Ethiopia [20,42,46], Turkey [44], and India [47] on the factors influencing early marriage and school dropout.

The study also examined the impact of education on early marriage and found that girls who had attended formal, primary, and secondary education were more likely to experience early marriage compared to those who had received higher education. This could be attributed to the fact that higher education levels increase girls' understanding of the implications of early marriage [1]. Maintaining girls' enrollment in schools has been demonstrated to postpone early marriage. Sekine and Hodgkin [48] in their study in Nepal, revealed that the likelihood of school dropout due to marriage increases after girls complete the fifth or sixth grade, reaching its peak during the seventh and eighth grades. This suggests that as girls advance through higher levels of education, they are more inclined to postpone marriage. Our bivariate modeling aims to account for this correlation within our interdependent outcomes.

Regarding media exposure, the study did not find a significant association between lack of access to mass media and school dropout. However, it is worth noting that limited access to media may lead to a lack of information and awareness about the importance of education, potentially contributing to higher dropout rates.

Finally, the study revealed that girls with uneducated partners were more likely to dropout from school compared to those with educated husbands or partners. This finding aligns with a study conducted in Nepal, suggesting that safety concerns, such as fear of rape and abduction, as well as restrictions imposed by husbands, particularly in rural areas with limited secondary school access, contribute to absenteeism and subsequent school dropout [48].

## 5 Conclusion

The results of this study provide evidence of a significant association between early marriage and school dropout among girls in Ethiopia. The bivariate binary multilevel logistic regression model employed in this study yielded superior results in predicting these outcomes. The study also revealed substantial variation in the prevalence of early marriage and school dropout among girls across different clusters in Ethiopia, with some administrative zones demonstrating a high risk for early marriage while others presented a high risk for school dropout after marriage.

The findings indicate that wealth index, decision to marry, residence, and auto-covariate were significant predictors for both outcomes, while girl's educational level, husband occupation, and ethnic group were significant predictors for early marriage. In contrast, media exposure and husband's educational level were significant predictors for school dropout. These results suggest that girls living in rural areas, those from the poorest wealth index, and those who did not receive formal education are more vulnerable to early marriage and school dropout than their counterparts.

Given the concrete evidence of the link between early marriage and school dropout among Ethiopian girls, it is imperative for policymakers and concerned entities to implement targeted interventions. Firstly, focusing on identified hotspot areas where the prevalence of early marriage and school dropout is notably high is paramount. These interventions should not only aim at delaying marriage but also at providing adequate support mechanisms to ensure girls remain enrolled in school. Furthermore, addressing the socio-economic and demographic factors highlighted in the study is crucial. Efforts should be made to improve access to education for girls residing in rural areas, especially those from economically disadvantaged backgrounds. This could involve implementing policies to provide financial incentives or scholarships to incentivize families to prioritize their daughters' education. Additionally, initiatives to enhance media exposure and educational opportunities for girls, particularly in rural communities, can play a significant role in reducing school dropout rates. Recognizing the significance of factors such as wealth index, decision-making autonomy, and educational attainment in determining girls' vulnerability to early marriage underscores the need for holistic interventions. Policymakers should consider implementing comprehensive strategies that empower girls economically, socially, and educationally. This might include initiatives to promote girls' education, improve economic opportunities for women, and raise awareness about the negative consequences of early marriage.

In addition to the recommendations outlined above, further research is warranted to deepen our understanding of the complex dynamics surrounding early marriage and school dropout among girls in Ethiopia. Future studies could explore the interplay between socio-economic factors, cultural norms, and educational policies to elucidate how these factors influence the likelihood of early marriage and subsequent school dropout. Additionally, qualitative research methodologies could be employed to gain deeper insights into the lived experiences of girls affected by early marriage and school dropout, allowing for a more nuanced understanding of the underlying drivers of these outcomes. Moreover, longitudinal studies tracking girls over time could provide valuable insights into the long-term impacts of early marriage on educational attainment and overall well-being. By expanding the scope of research on this topic, one can identify targeted interventions and policy strategies to effectively address the challenges faced by girls in Ethiopia and ensure their access to quality education and opportunities for a better future.

## 6 Limitations

It is important to note that the findings of this study may not fully represent the current situation in Ethiopia, given that the data used was obtained from the EDHS-2016 survey. Future research could incorporate more recent data, as well as explore additional cultural and behavioral factors that may impact the relationship between early marriage and school dropout. Moreover, it is important to acknowledge that interaction terms and random slopes were not incorporated in this study due to convergence issues encountered while estimating our model. While this limitation may not significantly impact the overall validity of our results, further exploration of these factors could offer additional insights into the intricate relationship between early marriage and school dropout in Ethiopia.

## Data availability statement

The data used for this study is publicly available at the DHS website, https://www.dhsprogram.com. However, the data that we generated will be made available up on request.

## CRediT authorship contribution statement

**Kassie Wubet Ebabu:** Writing – original draft, Formal analysis, Conceptualization. **Demeke Lakew Workie:** Validation, Supervision, Investigation, Conceptualization. **Ashenafi Abate Woya:** Supervision, Investigation. **Teshager Assefa Sisha:** Writing – review & editing, Supervision, Methodology, Investigation.

## Declaration of generative AI and AI-assisted technologies in the writing process

During the preparation of this work, the author(s) utilized *Claude AI primarily for addressing coding errors and conducting minor language editing*. After using this tool/service, the author(s) reviewed and edited the content as needed and take(s) full responsibility for the content of the publication.

## Declaration of competing interest

The authors declare that they have no known competing financial interests or personal relationships that could have appeared to influence the work reported in this paper.
